# Cadmium-Induced Pathologies: Where Is the Oxidative Balance Lost (or Not)?

**DOI:** 10.3390/ijms14036116

**Published:** 2013-03-18

**Authors:** Ambily Ravindran Nair, Olivier DeGheselle, Karen Smeets, Emmy Van Kerkhove, Ann Cuypers

**Affiliations:** Centre for Environmental Sciences, Hasselt University, Agoralaan Building D, Diepenbeek 3590, Belgium; E-Mails: ambily.ravindrannair@uhasselt.be (A.R.N.); olivier.degheselle@uhasselt.be (O.D.); karen.smeets@uhasselt.be (K.S.); emmy.vankerkhove@uhasselt.be (E.V.K.)

**Keywords:** cadmium, oxidative stress, antioxidants, organ toxicity, signalling, cancer, stem cells

## Abstract

Over the years, anthropogenic factors have led to cadmium (Cd) accumulation in the environment causing various health problems in humans. Although Cd is not a Fenton-like metal, it induces oxidative stress in various animal models via indirect mechanisms. The degree of Cd-induced oxidative stress depends on the dose, duration and frequency of Cd exposure. Also the presence or absence of serum in experimental conditions, type of cells and their antioxidant capacity, as well as the speciation of Cd are important determinants. At the cellular level, the Cd-induced oxidative stress either leads to oxidative damage or activates signal transduction pathways to initiate defence responses. This balance is important on how different organ systems respond to Cd stress and ultimately define the pathological outcome. In this review, we highlight the Cd-induced oxidant/antioxidant status as well as the damage *versus* signalling scenario in relation to Cd toxicity. Emphasis is addressed to Cd-induced pathologies of major target organs, including a section on cell proliferation and carcinogenesis. Furthermore, attention is paid to Cd-induced oxidative stress in undifferentiated stem cells, which can provide information for future therapies in preventing Cd-induced pathologies.

## 1. Introduction

Cadmium (Cd) is considered to be of major concern for public health by the World Health Organization [1]. Agricultural and industrial activities have led to the entry of Cd into the soil and subsequently into ground and drinking water. Due to the highly soluble nature of Cd compounds as compared to other metals, they are readily taken up by plants resulting in storage in crops for food and feed production [2]. This high soil-to-plant transfer rate makes the diet, in general, the primary source of Cd exposure in humans [3]. Vegetables and cereals are the main source of dietary Cd. A lesser percentage of Cd is found in meat products [4] and fish, except for crustaceans and molluscs that accumulate large amounts from contaminated aquatic environments [4]. Other sources of Cd exposure include smoking, occupational exposure and house dust [5]. Exposure to Cd via house dust is, besides the food, a significant entry route in areas with Cd-contaminated soils [4]. Cadmium is a major component of tobacco due to the hyperaccumulating characteristics of *Nicotiana tabacum*, which lead to high leaf Cd concentrations independent of the soil-Cd content [6]. In general, the Cd content in tobacco leaves ranges between 1 and 2 μg/g dry weight resulting in 0.5–1 μg Cd/cigarette. Furthermore, the Cd oxide generated during smoking either is deposited in lung tissues or absorbed into the systemic blood circulation of smokers [6]. This gives smokers 4–5 times higher Cd levels in blood and 2–3 times greater amounts of Cd in their kidneys than non-smokers [6]. Occupational exposure to Cd takes place in industrial factories such as zinc (Zn) smelters, battery manufacturing and metal recovering factories, Cd refining companies, paint and pigment production units as well as via other anthropogenic factors like waste incineration and fossil fuel combustion.

In addition to external factors, also intraspecies variation contributes to differences in the Cd body burden. Cadmium has a long biological half-time of 10–30 years in the human kidney, with women having a higher Cd body burden than men due to increased intestinal absorption of dietary Cd at low iron (Fe) stores [7]. Individual variations in Cd sensitivity and kidney Cd accumulation found in human population studies suggest that a considerable number of individuals may have toxic levels of Cd in their kidneys, despite the modest population mean values for Cd body burden [4]. Cadmium nephrotoxicity ensues at renal concentrations of ≥50 μg Cd/g wet tissue weight [4,7]. Although estimated dietary Cd intake varies widely in different countries [6], daily levels of Cd intake should be kept below 30 mg per person to be on a safer side [4].

The most important target organ for chronic low-level exposure to Cd is the kidney [8] and is reflected in proteinuria, calciuria, aminoaciduria, glycosuria and tubular necrosis [6]. Chronic low levels of Cd can lead to end-stage renal failure, deregulated blood pressure, diabetic complications and it also affects bone structure thereby leading to osteoporosis [6]. Chronic high levels of Cd exposure via oral ingestion, as was the case in Japan via contamination of rice fields, endemically led to the Itai-Itai disease. This contamination occurred as a result of irrigation with water polluted by Zn mine effluents located in the upper reaches of the Jinzu River basin [9]. The Itai-Itai disease is clinically characterized by tubular and glomerular dysfunction, and bone injury consisting of a combination of osteoporosis and osteomalacia [9]. Furthermore, Cd is also associated with airway inflammation [10], cardiovascular diseases [11], diabetes [12], neurological diseases [13] and several different organ cancers [14].

Information on the underlying molecular mechanisms of Cd-induced pathologies is rather fragmentary, however multiple studies indicate that Cd exposure induces oxidative stress at the cellular level [15]. Therefore, the current review focuses on the central role for oxidative stress as an underlying mechanism in Cd-induced pathologies. In this regard, pathologies in differentiated (organ toxicity) and non-differentiated cells are distinguished and discussed in relation to Cd-induced oxidative stress.

## 2. Cellular Mechanisms of Cd Toxicity: A Central Role for Oxidative Stress

The exact mechanism by which Cd is accumulated in cells remains vague, but it is considered that deregulation of transition metal homeostasis and use of cellular transport systems dedicated to essential elements contributes to the cellular uptake mechanisms of Cd [16,17]. It is hypothesized that Cd uptake involves competition with calcium (Ca), Fe and Zn and makes use of their transport systems [18–20]. Once taken up enterally, Cd reaches the liver where it binds to metallothioneins (MTs), glutathione (GSH) and other proteins or peptides [21]. Metallothioneins induced upon Cd exposure can act as a “double-edge sword”. On one hand MTs bind to Cd, thereby detoxifying and removing it from the cellular environment. On the other hand, due to its thiol groups, MTs can scavenge reactive oxygen species (ROS) that are produced as a result of Cd-induced oxidative stress [22]. However, the latter results in Cd dissociation from MTs due to the corresponding decreased metal binding stability [15,23]. Intracellular Cd, in bound or unbound form, culminates in mitochondrial damage, and/or cell death [21]. Cadmium interferes with mitochondrial oxidative phosphorylation and in higher doses can inhibit basal respiration [24]. It also affects the regulation of mitochondrial genes such as Hsp60 that play a role in cell protection and programmed cell death [24]. Different modes of cell death associated with Cd toxicity are dose-dependent and include necrosis, apoptotic-like cell death as well as autophagy exhibited by different cell types [25]. In brief, it seems that sub-micromolar concentrations of Cd lead to proliferation or delayed apoptosis, intermediate concentrations of 10 μM Cd cause various types of apoptotic cell death, and very high concentrations (>50 μM Cd) lead to necrosis [25].

**Figure 1:**
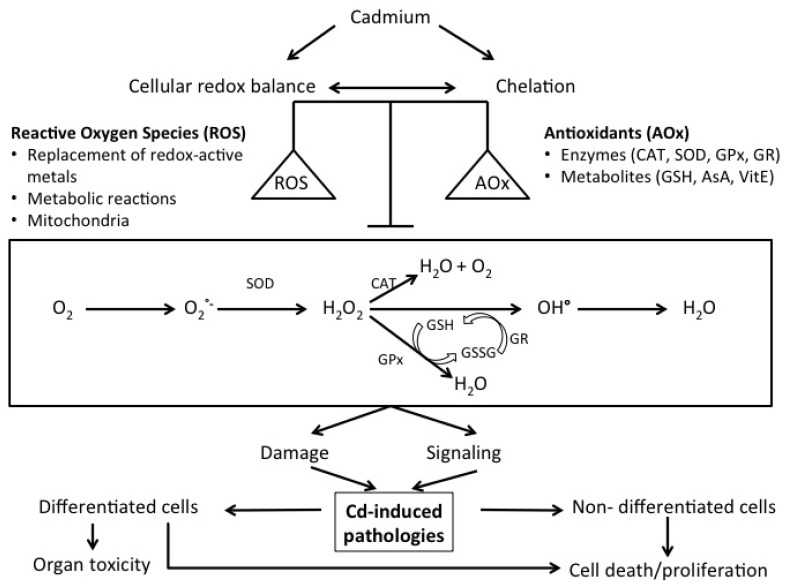
Simplified overview of the components involved in cellular Cd-induced oxidative stress. Reactive oxygen species (ROS); antioxidants (AO*_x_*); catalase (CAT); superoxide dismutase (SOD); glutathione peroxidase (GP*_x_*); glutathione reductase (GR); glutathione (GSH); glutathione disulphide (GSSG); ascorbic acid (AsA); vitamin E (VitE); superoxide (O_2_∘^−^); hydrogen peroxide (H_2_O_2_); hydroxyl radical (OH^∘^).

As Cd has no known useful function in humans, it evokes a number of cellular responses in which the cellular redox status plays a crucial role [15,26]. An overview on how Cd can disturb the redox balance is presented in Figure 1 and reviewed in Cuypers *et al.*[15]. In addition, it was demonstrated that oxidative stress could be part of early cellular responses affecting organ systems that ultimately lead to oxidative stress-induced pathologies (cfr. infra). It is therefore important to gather information on Cd-induced alterations on the cellular redox state and how this can lead to Cd-induced pathologies. As a non-fenton metal, Cd is unable to directly induce ROS [15]. However, indirectly, Cd induces oxidative stress by (1) a displacement of redox-active metals, (2) depletion of redox scavengers, (3) inhibition of anti-oxidant enzymes and (4) inhibition of the electron transport chain resulting in mitochondrial damage [15,21].

Several studies demonstrate the ability of Cd to replace Fe, a redox-active metal, thereby increasing the availability of free Fe in cells and hence inducing oxidative stress (Figure 1). As a redox-active element, Fe in its turn produces highly damaging hydroxyl radicals (∘OH) via the Fenton reaction [15,27]. Casalino *et al.*[27] demonstrated that in Fe-free conditions, lipid peroxidation, by means of TBARS (thiobarbituric reactive substrates) production, was absent in liposomes from male Wistar rats exposed to 75 μM CdCl_2_, indicating the inability of Cd to directly induce lipid peroxidation. On the other hand, TBARS production, induced upon Fe exposure alone (25 μM Fe^2+^), equalled that of lipid peroxidation induced by a combination of Cd and an Fe-containing (75 μM CdCl_2_ and 25 μM Fe^2+^) incubation medium. Also in rat Leydig cells, Cd-induced Fe displacement from its binding sites and consecutively Fe redistribution in these cells caused oxidative stress [28].

Cadmium also explores other ways to induce oxidative stress. As a thiol-affectionate metal, free Cd primarily targets the highly abundant cellular GSH, a ROS scavenger [15]. Depletion of the GSH pools leads to poor scavenging of Cd, which thereafter results in disturbance of the cellular redox balance leading to oxidative stress. Apart from antioxidant metabolites like GSH, antioxidant enzymes are also affected upon Cd exposure (Figure 1). Activities of superoxide dismutase (SOD), for example cytosolic CuZnSOD, can be differently altered to Cd intoxication depending on the duration of exposure. The activity of this enzyme is strongly inhibited by Cd when incubated for a short time (100–300 μM CdCl_2_ for 4 h) in contrast to its significant activation upon prolonged Cd exposure (100–300 μM CdCl_2_ for 8 h) in CRL-1439 normal rat liver cells [29]. This is also observed for catalase (CAT), glutathione peroxidase (GP*x*), and glutathione reductase (GR) activities that are known to increase or decrease depending on different experimental conditions [27,29–31]. Cadmium not only interferes with antioxidant defence mechanisms, also the mitochondrial electron transport chain is one of its main cellular targets. Cadmium mainly inhibits complex II (60%) and III (77%), whereas it can only weakly inhibit complex I (20%) and IV (30%) in mitochondria isolated from liver, brain and heart of male Dunkin-Hartley guinea pigs and exposed *in vitro* to different Cd concentrations [32]. The impairment of electron transfer through complex III by Cd may possibly be the route of ROS generation as Cd can bind to complex III resulting in accumulation of unstable semiubiquinones, which then transfer an electron to molecular oxygen, resulting in the formation of superoxide [32].

Although the complete pathology evoked by Cd toxicity is unknown, the ability of Cd to elicit an oxidative stress response seems apparent. Based on the fact that Cd-induced oxidative stress responses are dose, duration and tissue dependent [33,34], this review focuses on the main target organs of Cd-toxicity with special attention for the Cd-induced oxidative stress signature herein.

## 3. Cd-Induced Pathologies: A Central Role for Oxidative Stress

### 3.1. Kidney

Oxidative stress is an important mechanism underlying Cd-induced nephrotoxicity. In female Sprague-Dawley rats, a chronic exposure of 5 μmol CdCl_2_/kg body weight (subcutaneous injection), five days per week, lasting for up to 22 weeks showed that oxidative stress is a primary mechanism of chronic Cd-induced renal toxicity [35]. After 22 weeks, there was a 5.4-fold increase in TBARS renal levels, which could be reduced by co-treatment with antioxidants [35]. Cadmium exposure to primary culture of rat proximal tubular cells (1.25–40 μM CdAc_2_ for 12 h), demonstrated a concentration and time-dependent loss of cell viability (mostly apoptotic). Cytotoxicity was also observed in kidney tubular epithelial cells (Cos7) exposed to CdCl_2_ (0–80 μg/mL) for 24 h. This cytotoxicity was caused by Cd-induced oxidative stress and could be inhibited by antioxidant treatment of these cells with Propolis, a natural antioxidant product produced by honey bees [36]. The ability of the antioxidant *N*-acetylcysteine (NAC) to partially reverse apoptotic cell death implicates a definite role of oxidative stress in the apoptotic mechanism mediated by Cd [37]. The ROS production in these cells (at 2.5 and 5 μM CdAc_2_) can be the consequence of mitochondrial alterations as Cd-exposure induces a breakdown of the mitochondrial membrane potential. In a proximal tubular cell line, WKPT-0293 Cl.2, 5 μM Cd enhanced ROS production in 4–8 h. This further led to the degradation of Na^+^/K^+^-ATPase, a membrane protein that drives reabsorption of ions and nutrients through Na(+)-dependent transporters in the proximal tubules, via proteasomal and endo-/lysosomal proteolytic pathways [38]. This, in its turn contributes to the 'Fanconi-like syndrome’ in which Na^+^-dependent transport is diminished and is associated with Cd-induced nephrotoxicity [38]. Furthermore, in these cells treatment with antioxidant agents such as NAC and pyrrolidine dithiocarbamate (PDTC), prevented ROS induction after Cd exposure (5 μM CdCl_2_ for 4–8 h). Increased ROS levels in WKPT-0293 Cl.2 cell lines induced the gene expression of the multidrug resistance transporter gene (mdr1) by a process involving NF-κB activation. This overexpression of mdr1 protects proximal tubule cells against Cd-mediated apoptosis [39]. Mitochondria also play a crucial role in Cd-mediated proximal tubular toxicity [40]. An intraperitonial injection of 0.3 mg Cd as CdMT/kg body weight to Sprague-Dawley rats resulted in Cd accumulation in mitochondria, resulting in mitochondrial swelling, electron transfer inhibition as well as oxidative phosphorylation [41].

### 3.2. Liver

It has been shown that a daily oral administration of a low dose Cd (4.4 mg CdCl_2_/kg) via drinking water during 120 days in female Sprague-Dawley rats resulted in the formation of ROS, which enhanced hepatic lipid peroxidation and nuclear DNA damage [42]. Cadmium-induced lipid peroxidation in the liver could be counteracted by supplementation of vitamin E in rabbits [43], where white rabbits were given tap water with or without Cd (1 g CdCl_2_/L), or tap water containing CdCl_2_ plus vitamin E (100 mg/dL α-tocopheryl acetate in 0.2 mL corn oil) on a daily basis for 30 days. Liver protection by pre-treatment with antioxidants (heated garlic juice and ascorbic acid; each 100 mg/kg body weight for 4 weeks) was also demonstrated in adult male Wistar rats. Whereas the rats received 4 mg/kg body weight CdCl_2_ for three days at the last week of antioxidant treatment, lipid peroxidation could be significantly decreased [44]. Administration of 10 mg Cd/L (as Cd acetate) to Wistar rats during gestation and lactation also increased lipid peroxidation and CAT activity in pup liver, and is highly hepatotoxic to pups from the first day of birth on [45]. Looking at the antioxidative defence system, it was demonstrated that Cd exposure resulted in GSH depletion in rat liver when male albino rats were intraperitoneally exposed to 0.1 mg and 1 mg CdCl_2_ /body weight for three months (five days/week) [46]. The authors argue that the depletion of GSH at a low dose (0.1 mg CdCl_2_/kg body weight) might be due to differences in dose, route of exposure and long duration while the GSH depletion at higher dose (1 mg CdCl_2_/kg body weight) occurs due to ROS. In addition to antioxidant metabolites, antioxidative enzymes are also affected by Cd stress. Intraperitoneal administration of 0.4 mg Cd/kg weight to male albino rats for 45 days inhibited GPx and CAT activities in liver [47]. Activities of SOD, CAT, glutathione reductase (GR) and GPx were diminished in a normal rat liver cell line, CRL-1439, upon a 4 h exposure to Cd ranging from 100 to 300 μM CdCl_2_[29]. In these cells, more oxidative stress was observed in mitochondria rather than in cytoplasm and depending on different concentrations of Cd (0–150 μM CdCl_2_ for 24 h), antioxidant enzymes were activated or inhibited. A CAT assay was performed separately on mitochondrial (mit) and cytoplasmic (cyt) extracts of Cd-treated cells, which showed an increased mit-CAT activity of 60.3%, 88.0%, and 80% to 50, 100 and 150 μM of CdCl_2_ respectively while an increase in cyt-CAT activity was restricted to 10.4% and 50.5% at 50 and 100 μM CdCl_2_[48]. A further increase in dose to 150 μM CdCl_2_ decreased the cyt-CAT activity to the untreated control levels. This was also true for GR activity, which increased in mitochondria and cytoplasm at 50 μM CdCl_2_, but drastically decreased in mitochondria at 100 and 150 μM. The cyt-GR activity was the highest at 100 μM CdCl_2_. It appears that mitochondrial enzymes were more effective in reducing various ROS than their cytoplasmic counterparts and the activities of antioxidant enzymes in the cytoplasm were not as high as the mitochondrial enzymes upon Cd treatments [48]. Although the mitochondrial antioxidant system is very effective, Cd-induced ROS production in mitochondria is strongly associated with cell death. In a human hepatocarcinoma cell line, Hep3B, Cd exposure (2.5–10 μM, 48 h) induced apoptosis independently of caspase activation through a mechanism involving nuclear translocation of 2 mitochondrial proteins, endonuclease G (involved in induction of caspase-independent DNA fragmentation) and apoptosis-inducing factor (AIF) [49]. This study showed that the release of these mitochondrial proteins was closely associated with massive ROS production, which resulted in alteration of mitochondrial homeostasis leading to calcium (Ca)-induced dissipation of mitochondrial membrane potential as well as decreased expression of anti-apoptotic bcl-x_L_ protein regulated by NF-κB [50]. While electron spin resonance studies on ROS detection has shown a minimal role for ROS in chronic Cd hepatotoxicity [50], it provides direct evidence of involvement of ROS in acute exposure conditions *in vitro* and *in vivo*[50–52].

### 3.3. Bone

Extensive epidemiological studies provide repeated evidence of increased Cd exposure correlating significantly with decreased bone mineral density (BMD) and increased fracture incidence at lower exposure levels of Cd [53]. Cadmium is also negatively associated with bone mineral density in post-menopausal women [54] and a relation between the oxidative/antioxidative status, and bone mineral density (BMD) and fracture rate was noted in osteoporotic patients [55,56]. Even though the first epidemiological argument for Cd-induced bone effects was the clear-cut interference of low level Cd exposure with Ca metabolism [54], there are only a few studies that imply oxidative stress as a mechanism for Cd-induced osteotoxicity [56,57]. Smith *et al.* proved *in vitro* in an osteosarcoma cell line, Saos-2, using 5–50 μM CdCl_2_ for 3–48 h that Cd-induced oxidative damage led to a decrease in RUNX2 expression resulting in osteoblast apoptosis suggesting RUNX2’s anti-apoptotic role in osteoblasts. RUNX2 is an osteoblast transcription factor, which is known to play a protective role against osteoporosis in postmenopausal women [57]. A protective role of macrophage migratory inhibitory factor (MIF) was also demonstrated in murine osteoblast MC3T3-E1 cell lines. In these cell lines, noncytotoxic concentrations of Cd (0–1 μM CdCl_2_ for 24 h) induced an upregulation of this factor [58]. It is thought that Cd-induced ROS results in NF-κB activation that subsequently enhances the transcription of the MIF gene and other protective target genes [58]. *In vivo* studies by Brzoska and colleagues showed that Cd (5 or 50 mg Cd/L), when fed to male Wistar rats in drinking water for six months, weakened the antioxidative capacity of the bone tissue and led to oxidative stress [56]. There was increased lipid peroxidation and H_2_O_2_ production as well as decreased activities of GPx, SOD and CAT. The accumulated ROS and oxidised lipids may affect the metabolism of bone tissue and these Cd-induced changes in the bone oxidative/antioxidative status can lead to disorders in the bone marrow turnover and mineralization. It was shown that delicate interactions between nitric oxide, ROS and antioxidant enzymes take place in the process of bone loss in post-menopausal women [55].

### 3.4. Lungs

The lung is also considered as one of the target organs of Cd toxicity. Cadmium enters the lung via house dust, smoking and/or occupational exposure (cfr. supra) [5]. Cadmium can induce apoptosis in rat lung epithelial cell lines and a possible underlying mechanism is the induction of ROS. This conclusion is based on the fact that exposure of these cell lines to 20 μM CdCl_2_ during 24 h resulted in a 4-fold increase of the oxidized GSH pool (glutathione disulphide: GSSG), thereby altering the GSH homeostasis. Cadmium (10–50 μM CdSO_4_ for 1–3 days) is known to decrease the expression of cystic fibrosis transmembrane conductance regulator (CFTR) protein in human airway epithelial (Calu3) cells and subsequent decrease of chloride transport in the cell [59]. The antioxidant α-tocopherol was able to prevent the loss of this protein indicating a role for oxidative stress. This protein is also responsible for GSH secretion to protect lung tissue against damage [60] and any mutation in this protein can result in low GSH levels in the cell leading to an oxidative stress environment [61]. Apoptotic concentrations of Cd (0–30 μM CdCl_2_, 0–72 h) led to (1) the upregulation of antioxidative genes like glutathione-S-transferase-α (GST-α), γ-glutamylcysteine synthetase (γ-ECS; 1st biosynthetic enzyme in GSH synthesis) and MT 1 and (2) also augmented the DNA binding activities of redox-regulated transcription factors like AP-1 and NF-κB [62]. However, another study in primary cultures of epithelial cells, like alveolar type 2 cells and Clara cells, isolated from rat lung showed that the Cd-induced apoptosis (1–10 μmol/L for 20 h) was Bax and p53 dependent, but was independent of oxidative stress pathways [63]. This apparent contradiction can be explained by the different exposure conditions (time and dose), but needs further investigation.

Smoking is known to cause chronic obstructive pulmonary disease (COPD) in 90% of the smokers and is characterized by chronic airway inflammation and airflow limitation [10,64]. The components of tobacco smoke, aside from nicotine, such as heavy metals and carcinogens can lead to an oxidative stress environment [65]. Cadmium, a major constituent of cigarettes has proven to cause pulmonary oxidative stress, emphysema and persistent airway inflammation in rat models that mirror the conditions observed in COPD patients [10]. Sprague-Dawley rats that received nebulised Cd via inhalation (0.1% CdCl_2_ in 0.9% NaCl) during a single exposure of 1h showed acute increase of GSSG in their bronchoalveolar lavage fluid (BALF), which was balanced by simultaneous increase of GSH. Animal groups that underwent repeated exposure to Cd (1 h for 3–5weeks) showed progressive increase of BALF-GSH, which is in agreement with findings observed in COPD patients [10]. The universal antioxidant transcription factor Nrf2 has been recently implicated in broad range of responses involved in both the initiation and progression of lung injuries caused by smoking [65]. Mice lacking Nrf2, when exposed to cigarette smoke (7 h per day, 7 days per week, during six months) were more susceptible to emphysema, had elevated levels of alveolar DNA oxidation as well as enhanced alveolar oxidative stress that regulates the intensity of alveolar cell inflammation and apoptosis [66].

### 3.5. Cardiovascular System

Cadmium is an independent novel risk factor for cardiovascular diseases (CVDs) and induces CVDs *in vitro* and *in vivo*[11,67]. The exact role of Cd in CVDs is controversial [68], but it can alter endothelial gene expression and lead to patho-physiological changes at low levels of exposure [11,69]. Oxidative stress induced by Cd might be one of the reasons for cardiovascular effects as low-density lipoprotein (LDL) modification by oxidative damage is a key event in development of atherosclerosis and oxidized LDL particles are found in atherosclerotic lesions [68,70]. Male Buffalo rats that were given 50 or 200 ppm Cd in drinking water exhibited increased lipid peroxides and GSH. It also increased arterial blood pressure and blunted the vascular responses to vasoactive agents [69]. Donpunha *et al.* demonstrated the presence of oxidative stress in Cd-induced hypertension and vascular dysfunction, when a subchronic dose of Cd (100 mg CdCl_2_/L in drinking water) was supplied to male ICR mice for 8 weeks [71]. The oxidative damage was alleviated by supplementation of ascorbic acid (50 or 100 mg/kg body weight), possibly by suppressing ROS formation and maintaining the GSH pool and hence, redox balance [71]. Low concentrations of ZnCl_2_ (10 μM) could also significantly inhibit Cd-induced ROS production and apoptosis caused by exposure of bovine endothelial cells, isolated from calf aorta, to 0.1–100 μM CdCl_2_ for 24 h [72]. The mechanism suggested for the inhibition of apoptosis is the ability of Zn to inhibit Ca^2+^-dependent endonuclease activity. Thereby DNA fragmentation, which is the terminal step in apoptosis, is inhibited. However, the authors could not exclude that the inhibition of apoptosis might have been caused by a decreased accumulation of intracellular Cd, when applied together with Zn.

### 3.6. Brain

Cadmium cannot penetrate the adult blood brain barrier (BBB), although it might diffuse across the BBB with the help of a vehicle such as ethanol [73]. Cadmium can more effectively pass the BBB during the developmental stage in an organism and is more toxic in newborns [74,75]. Once inside, it accumulates in different areas of the brain, induces lipid peroxidation and weakens the antioxidative defence [74,76]. In battery workers Cd-induced oxidative stress was demonstrated to cause amyotrophic lateral sclerosis due to reduced brain SOD activity [77]. Cadmium (0.4 mg CdAc_2_/kg body weight) injected intraperitoneally to young albino rats for 30 days generated free radicals in the brain causing region-specific membrane changes, which in turn led to significant alterations in membrane fluidity, intracellular Ca concentrations and phospholipid composition [78]. It also resulted in a decreased GSH/GSSG ratio as well as activities of GR and glucose-6-phospate-dehydrogenase (G6PDH) in various brain regions, although the decrease in GSH/GSSG was not seen in the hippocampus and midbrain [79]. Cadmium-induced oxidative damage also induced enhanced lipid peroxidation and protein carbonylation in male Swiss albino mice that received 4 mg CdCl_2_/kg body weight orally for three days [74]. The oxidative impairment was characterised by increased ROS production, reduction of total thiols and the GSH pool together with an increase in GSSG level. In addition, also activities of antioxidant enzymes such as SOD, CAT, GST, GR, GPx and G6PDH were diminished. The authors also showed the protective abilities of taurine (single oral dose of 100 mg/kg body weight for five days before Cd treatment) and vitamin C (single oral dose of 100 mg/kg body weight for five days before Cd treatment) against oxidative impairment in brain tissue caused by Cd. Furthermore, vitamin C was also demonstrated to reverse Cd-induced apoptotic cell death in cortical neurons, while necrotic cell death remained unaltered. This confirms the involvement of ROS in apoptosis [31]. In a mouse neuroblastoma cell line HT4, it was shown that cell death mechanisms and pro-inflammatory responses induced upon Cd exposure are redox-dependent. Cd-induced oxidative responses could be reversed in these cells, when treated with NAC and COX-2 inhibitor celecoxib. COX2 activation is necessary for Cd-induced pro-inflammatory responses and is mediated by a signalling cascade comprising of PI3K (phosphatidylinositide 3-kinase), a flavoprotein and p38 MAPK (mitogen-activated protein kinase) [80].

Whereas Cd exposure clearly leads to oxidative damage, it has been shown that Cd-induced ROS generation (10–20 μM CdCl_2_) in rat pheochromocytoma (PC12) and human neuroblastoma (SH-SY5Y) cells resulted in activation of signalling pathways such as JNK, Erk1/2, p38 MAPK and their upstream kinases like ASK1, MKK4, MEK1/2, and MEK3/6 leading to caspase-dependent and independent apoptosis. Pre-treatment with NAC remarkably inhibited Cd-induced phosphorylation of these kinases [81] and the authors suggest the use of antioxidants as well as inhibitors of JNK or ERK1/2 to be exploited for prevention of Cd-induced neurodegenerative diseases. Sustained phosphorylation of these stress-activated kinases, JNK and p38 MAPK, and their downstream targets, c-Jun, ATF-2 and CREB were also observed in a mouse neuroblastoma cell line, HT4, in response to Cd (3–30 μM CdSO_4_ for 24 h) characterised by increased ROS production and heme oxygenase-1 (HO-1) induction [82]. The variable effects of Cd on oxidative stress signature in different experimental set-ups are also reported in brain cells [83].

### 3.7. Testis

Exposure of Swiss mice to CdCl_2_ (1 mg/kg body weight) for 5–8 weeks, increased testicular lipid peroxidation, thereby impairing intracellular defences leading to altered spermatogenesis [84]. A significant reduction in enzymatic activities of SOD, GPx as well as CAT was observed in these cells together with a decline in ascorbic acid content [84]. Supplementation of vitamin C and E could ameliorate testicular stress to a certain extent. Also in Cd-exposed rats an increase in testicular lipid peroxidation and a decrease in the antioxidant enzyme activities, such as GPx and SOD, were observed. Pretreatment with vitamin C and E reduced testicular ROS production, thereby restoring normal testicular function [85]. In addition, the nutritional antioxidant beta-carotene alleviated oxidative stress and loss of antioxidants in adult male rats intragastrically exposed three days a week to 2 mg CdCl_2_/kg body weight during3–6 weeks [86].

The high membrane lipid content of testicular Leydig cell mitochondria and microsomes makes these cells more susceptible to Cd-induced lipid peroxidation [87]. Testicular Leydig cells are also the target cell population for Cd carcinogenesis. A single carcinogenic dose of CdCl_2_ (30 μmol/kg body weight) caused severe hemorrhagic damage in rat testis within the first 12 h after exposure together with increased Fe content, H_2_O_2_ production and lipid peroxidation [28]. At this point, GSH levels were decreased, concomitantly with a rise in GSSG levels. Also GPx activity was increased, GR and CAT were reduced and SOD remained unaltered. Atrophy with calcification occurred in 2–3 months and atrophied tissues were regenerated towards the end of 1 year after exposure. The authors concluded that the Cd doses that compromise cellular defence mechanisms and hence induce oxidative stress, may have an important role in the initiation of carcinogenesis within the target cell population.

## 4. Cancer

**Figure 2:**
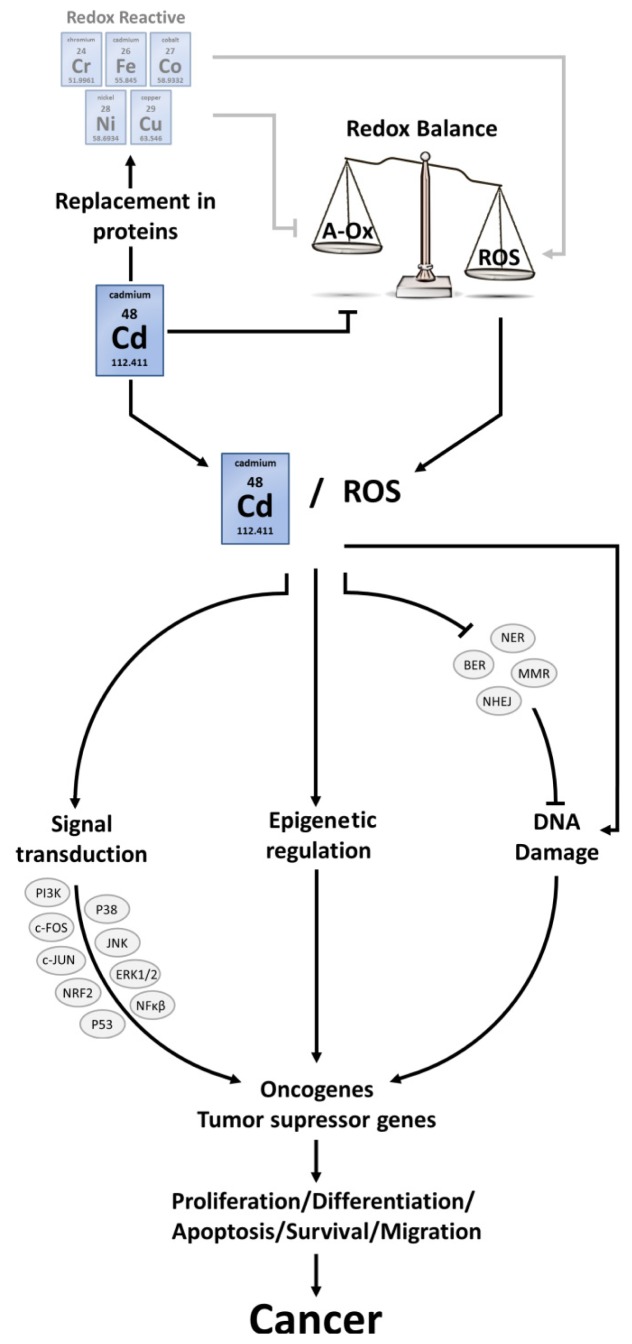
Schematic overview of Cd-induced carcinogenesis. Reactive oxygen species (ROS); nucleotide excision repair (NER); base excision repair (BER); mismatch repair (MMR); non-homologous end-joining (NHEJ); phosphatidylinositide 3-kinases (PI3K); mitogen activated protein kinase p38 (P38); c-Jun N-terminal kinase (JNK); nuclear factor (erythroid-derived 2)-like 2 (NRF2); extracellular-signal-regulated kinases (ERK); tumour protein 53 (P53); nuclear factor kappa-light-chain-enhancer of activated B cells (NFκβ).

Cadmium is classified by the International Agency for Research on Cancer [88] and by the US National Toxicology Program [89] as a human carcinogen. As such, the negative effect of Cd on pulmonary tumour formation was indicated in both epidemiological and experimental studies [14,90,91]. There is also clear evidence of cancer progression due to Cd toxicity within the prostate [92–94], kidney [95,96], breast [97,98], endometrium [99], bladder [100–103] and pancreas [14,104–106]. Evidence for the involvement of Cd in the development of stomach, liver and hematopoietic cancers, however, is not very convincing [14,107]. Cadmium-induced carcinogenesis is a well-discussed topic recently reviewed by several independent researchers [25,107–112]. Both, oxidative stress and inhibition of repair of oxidative DNA damage undoubtedly influence this process, although some papers state that the role of ROS in the process of cancer formation is minimal [50,113]. Several studies discuss the impact of induced adaptation mechanisms upon chronic Cd exposure, where diminished ROS levels were detected as a result of increased antioxidant levels such as GSH and MTs [22,50]. As a consequence, a condition of increased apoptotic resistance is created where DNA damaged cells can escape from elimination through apoptosis, and proliferate with inherent DNA lesions, eventually progressing to a malignant phenotype. Nevertheless, multiple studies indicate Cd-induced ROS formation, which affect different pathways in the development of malignancies, thereby inducing or strengthening Cd-provoked carcinogenesis. Within the perspective of the current review, carcinogenic processes will be highlighted in function of their sensitivity to redox disturbances induced by Cd based on relevant literature that supports the role of ROS in the formation of cancer tissue (Figure 2).

### 4.1. ROS Interconnect with Signalling Pathways

Both Cd and ROS interfere with the activation of oncogenes, the inhibition of tumour suppressor genes and influence signal transduction processes via the modulation of transcription factors. More specifically, Cd replaces Zn in Zn-binding domains while ROS attack thiol groups of cysteine residues. One of the affected pathways involves the activation of c-fos and c-jun transcription factors, which together form AP-1. This transcription factor is responsible for the activation of proto-oncogenes involved in cell growth and division [114]. Tumour formation in BALB/c-3T3 cells (an established fibroblast cell line from albino mice), exposed to 6 and 12 μM CdCl_2_ for 72 h, was accompanied by higher expression levels of c-fos and c-jun [115]. The same effects were seen in the rat liver epithelial cell line TRL 1215, exposed to 1 μM CdCl_2_ up to 28 weeks [113].

Another signal transduction pathway that links ROS with carcinogenic processes is the mitogen activated protein kinase (MAPK) pathway. In human neuroblastoma cell lines, an exposure to 10 and 20 μM of CdCl_2_ for 24 h led to an increased activity of c-Jun N-terminal kinase (JNK) and extracellular-signal-regulated kinases (ERK) after ROS-induced disruption of serine/threonine phosphatases 2A and 5 [81]. Both JNK and ERK1/2 are regulators of apoptosis and cell proliferation and contribute in the transition of cells to cancer [111]. However, care should be taken, as Cd-induced redox alterations not always provoke identical responses concerning the MAPK cascade. In human prostate epithelial cells (RWPE-1) chronically exposed to 10 μM of CdCl_2_, for example, the induction of cancer was accompanied by disruption of the JNK pathway via Bcl2 overexpression [116].

Also the phosphatidylinositide 3-kinases (PI3K) pathway, responsible for inhibition of apoptosis and stimulation of proliferation through protein kinase B (AKT) and mammalian target of rapamycin (mTOR), is affected after Cd exposure in a ROS-dependent way. In chronically exposed human bronchial epithelial BEAS-2B cells to 2 μM Cd (for 2 months), the induction of cell transformations into cancer cells was linked to the induction of AKT [117], an effect that diminished after antioxidant treatment (transfection with CAT, SOD1 or SOD2). Both ERK and AKT interact with ROS signalling in immortalized human lung epithelial BEAS-2B cells and normal human bronchial epithelial cells exposed to 5 μM of CdCl_2_ for 4 h, thereby inducing the expression of the proangiogenic molecule hypoxia-inducible factor-1 (HIF-1). HIF-1 is a promoting factor in tumour formation.

Cadmium also interferes with the (de)activation of other signalling proteins such asp53, NRF2 and NF-κβ [112,118]. They are involved in maintaining the balance between proliferation and apoptosis and when disrupted by Cd, hyperproliferation or apoptotic resistance can be induced. The involvement of ROS herein, however, still remains to be elucidated.

### 4.2. ROS-Induced DNA Damage

DNA damage generated by Cd-induced ROS is not easily repaired, as Cd interferes with all DNA repair systems, among which are nucleotide excision repair (NER), base excision repair (BER), mismatch repair (MMR) and non-homologous end-joining (NHEJ) [112,119]. A lot of proteins involved in DNA repair systems have Zn-binding proteins that can directly be disrupted by Cd [107,112]. However, there is also a link between Cd-induced ROS and the inhibition of DNA repair. Critical cysteine residues on 8-oxoguanine DNA glycosylase (OGG1), one of the compounds of the BER system, can be indirectly oxidized by Cd, thereby inhibiting proper functioning of this enzyme [120,121].

The link between Cd-induced ROS and DNA damage has been accounted to the formation of 8-hydroxy-2′-deoxyguanosine (8-OHdG), a critical marker for oxidative stress and carcinogenesis [122,123]. An association between Cd and the formation of 8-OHdG was also seen in glass production workers [124]. On the other hand, the link between the progression of cancer tissue and the presence of 8-OHdGs has been proven in both human and animal tumour models [125–127]. These data combined could give an indication that the formation of 8-OHdGs as a result of Cd-induced ROS formation is an important element in the progression of cells towards cancer.

### 4.3. ROS and Epigenetic Alterations

The epigenetic state of the genome determines the gene expression profile of an organism without changing the DNA sequence, and is determined by the function of different proteins such as DNA methyltransferases (DNMTs), histone deacetylases (HDACs), histone acetylases, histone methyltransferases and the methyl-binding domain protein MECP2 [128]. Cadmium interferes with the epigenome, thereby changing gene expression profiles in favour of carcinogenesis [129]. In chronic myelogenous leukemia (K562) cell lines exposed to 2 μM of CdCl_2_, global DNA hypomethylation was associated with Cd-stimulated cell proliferation [130]. Also in rat liver cells exposure up to 500 μM CdCl_2_ led to an increased activity of DNA methylation proteins (DNMT) [131]. In immortalized normal human prostate epithelial cells exposed to 10 μM of CdCl_2_ for 10 weeks, the overexpression of DNMTs and genomic hypermethylation were associated with Cd-exposure [132]. In human bronchial epithelial cells (16HBE) exposed to CdCl_2_, DNMT genes were overexpressed which resulted in global DNA hypermethylation [133]. Either way the result is hypomethylation or hypermethylation of respectively oncogenes and tumour suppressor genes, which could induce altered gene expression patterns that lead to carcinogenic events.

Huang *et al.*[130] tested if the changes in global DNA methylation during Cd exposure could be accounted to elevated ROS levels. Elimination of ROS via NAC did not reset the global DNA methylation changes. This could indicate that hypomethylation or hypermethylation is the result of direct interference by Cd and not ROS [130]. However direct exposure to ROS (without the involvement of Cd) has shown to induce epigenetic changes as well, so mechanisms through Cd-induced ROS can still apply [134,135].

## 5. Stem Cells

In the previous part, we discussed how the redox balance contributes to the transition of normal cells to cancer cells. Carcinogenic processes (1) can be initiated in specialized cells, which often result in dedifferentiation, or (2) can start in undifferentiated cells [136]. Undifferentiated cells or stem cells are characterized by their high capacity of self-renewal and differentiation. They are highly resistant to many stressors such as chemical compounds, ultraviolet light, radiation and oxidative stress [118,137], a property that makes them unique for studying cellular maintenance and protection. Stem cells wield two main defence strategies, quiescence and damage control, which are discussed below in function of their responses to redox-related changes.

### 5.1. Defence Mechanisms in Stem Cells

Quiescent cells are cells that are kept in a G_0_ resting phase, a process that is critical to preserve successful self-renewal [138,139]. The defence strategy of quiescent cells resides in the fact that they have a low metabolic status, a high efflux capacity (of cytotoxic compounds) through ATP-dependent transporters such as MDR1 [116] and an extensive network of scavengers [118,137,140]. Moreover, quiescence is characterized by a strict regulation of the redox balance in which ROS levels are kept low [118,137]. In some conditions, damage is inevitable, and damage control mechanisms are activated. Depending on the level and type of damage inflicted, stem cells can repair damaged DNA, drive the cells into cellular senescence or induce apoptosis [118,137,141].

### 5.2. Cadmium and Stem Cells

**Figure 3:**
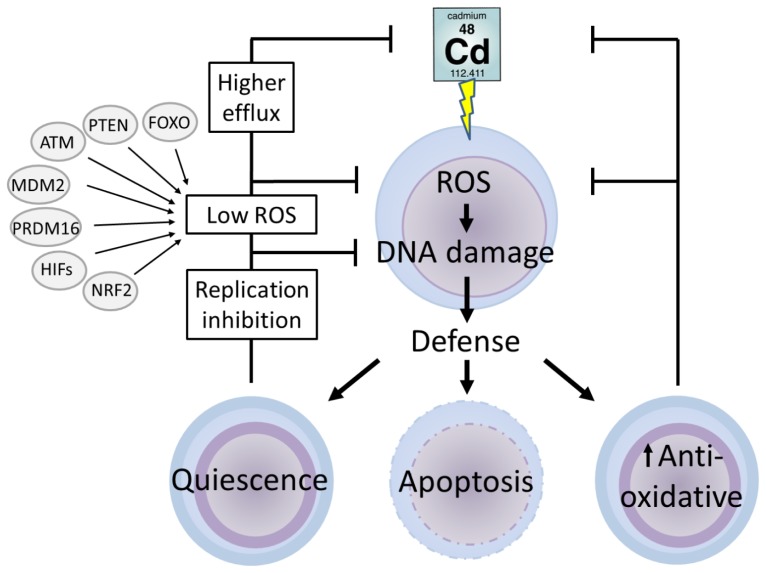
Schematic overview of Cd toxicity in stem cells in general. Intoxication of stem cells by Cd could indirectly induce oxidative stress by impairment of the redox balance. The excess of reactive oxygen species (ROS) can induce DNA damage. The reaction of stem cells to Cd-induced toxicity is ambiguous. Increased levels of ROS can either be removed directly through induction of antioxidative mechanisms or indirectly through induction of the quiescent stem cell model. Quiescence will keep the level of ROS generation low by signalling through FoxO transcription factor (FOXO), phosphatase and tensin homolog (PTEN), ataxia telangiectasia (ATM), murine double minute oncogene (MDM2), PR domain-containing 16 (PRDM16), hypoxia inducible factors (HIFs) and nuclear factor erythroid-2-related factor 2 (NRF2). On the other hand the increased levels of ROS will trigger signalling cascades that induce apoptosis to prevent the accumulation of damaged stem cells by ROS.

Stem cells are designed to maintain low levels of ROS [142]. Within these cells, Cd will raise the levels of ROS concomitantly with several defence mechanisms. Despite the limited data available on this topic, a few strategies of stem cells coping with Cd-induced oxidative stress are hypothesized (Figure 3). On one hand, increased levels of ROS can be removed directly through activation of anti-oxidative mechanisms. On the other hand, ROS levels are indirectly controlled via signalling mechanisms during the quiescent stem cell mode, to maintain low ROS levels. Examples of these regulators are PTEN [143,144], ATM [145,146], MDM2 [147], PRDM16 [148], HIFs [149,150], FOXO [151,152] and NRF2 [153,154]. A third way of responding is the induction of apoptosis to prevent an accumulation of damaged stem cells.

Cadmium induces ageing-like effects via oxidative stress, a process that was counteracted in murine fertilized zygotes after treatment with antioxidants [155]. A direct removal of Cd-induced ROS was also observed in alveolar type II epithelial stem cells (that are able to differentiate into type I cells) [156]. The levels of both, MT and GSH, were strongly induced when these cells were exposed to CdO aerosols (1.6 mg Cd/m^3^) for 5 to 7 weeks [157]. The impact of (antioxidative) defence mechanisms in the coping strategy of a stem cell was also demonstrated in the pluripotent stem cells of planaria (organisms capable of extreme regeneration). Planarian stem cells, also known as neoblasts, showed an increased expression of heat shock proteins (HSP60 and HSP70) when exposed to 2.5, 5 and 10 μM of CdCl_2_ from two days up to 1 week [158]. In this manuscript, the authors hypothesize an important role for HSPs in the stem cell defence, guiding survival and proliferation during Cd stress. The involvement of other antioxidative enzymes important in somatic cell defence [15], is not yet clear for Cd-exposed undifferentiated cells. However, based on the studies described above, a degree of similarity can be concluded.

Overall, among different types of stem cells, an increased number of quiescent cells is observed during Cd stress. As such, the number of divisions and the grade of differentiation was strongly inhibited in murine embryonal carcinoma cells exposed to Cd [159], in murine embryonic stem cells (mESC) exposed to 110 μM CdCl_2_ for 1 h [160] and in mESCs exposed to cigarette smoke or cigarette smoke condensate for 4 weeks [155]. A similar inhibition of self-renewal and proliferation was also observed in prostate stem progenitor cells exposed to 450 μM CdCl_2_ for 8 weeks [161]. Also for the pluripotent stem cells of the planarian *Polycelis felina* mitotic activity was decreased when exposed to 1, 10 and 100 μM of CdSO_4_[162]. All these (temporary) proliferation stops indicate that a large amount of stem cells goes into a quiescent mode or dies to protect them from further Cd-induced stress. A decrease in cell proliferation not only coincides with cell death, but is also associated with the induction of cell differentiation. As such, undifferentiated neural precursors were forced into astate of active differentiation after exposure to Cd [163] and references herein). All of these findings are in contrast with the number of mitotic divisions measured in the planarian *Schmidtea mediterranea*, which increased when exposed to 2.5, 5 or 10 μM of CdCl_2_ for at least two weeks [158]. This result is not totally unexpected since the stem cells of these organisms are pluripotent and powerful towards stress and ageing [164,165]. However the elevation in mitotic divisions can also be explained by the fact that the cells were measured via histochemical visualization in the M-phase, which could indicate that dividing cells still need to enter the G_0_ phase. Contradictory responses on stem cell proliferation after Cd exposure were also observed in neural precursor cells, an effect that appeared to be concentration-dependent [163]. This corresponds with somatic cell responses, where increased proliferation during low Cd exposure is often classified as an hormesis effect [166–170].

When defence strategies fail, damage is inevitable, even in stem cells. In mesenchymal stem cells, an exposure to 15 and 45 μM of CdCl_2_ for 24 or 48 h led to an elevated level of DNA damage which led to nuclear breakage and chromatin condensation [171]. This effect was confirmed in mESCs during both acute and chronic exposure to 20 μM of CdCl_2_[172]. Damage to DNA strongly affects normal function or even viability of cells, resembled by telomere shortening during chronic exposure of mESCs to 20 μM of CdCl_2_[172]. A cellular defence mechanism against extreme (molecular) damage is apoptosis. In mESCs the exposure to 20 μM of CdCl_2_ for 1 h led to the activation of JNK through phosphorylation of MKK4 and MKK7, which led to the induction of apoptosis [173]. In Hela cells exposed to 50 μM of CdSO_4_ for 6 h the intrinsic pathway of apoptosis was induced, while in contrast the extrinsic pathway of apoptosis was inhibited, but in the end it led to cell death [174].

Genomic instability not only induces cell death, but as described earlier, Cd-induced genomic instability also leads to neoplastic transformation. Evidence for stem cells involved in Cd-induced cancer, was reported by Hart *et al*[157]. They showed that Cd inhibits DNA repair in alveolar epithelial stem cells exposed to CdO aerosols (1.6 mg Cd/m^3^) for 5 to 7 weeks, which led to neoplastic transformations. Also the reprogramming and transformation of prostate stem cells and early stage progenitor cells into cancer cells by Cd was reviewed recently [175]. Nevertheless, more information is needed to further elucidate a clear role for stem cells in Cd-induced carcinogenesis.

Overall, stem cell responses to Cd stress are ambiguous. Thanks to their extensive defence strategies, among which quiescence, damaging processes can be overcome more easily. If damage does occur and accumulates after Cd intoxication, stem cells can be triggered into apoptosis. Cadmium-induced damage such as genomic instability, however, is not always re-balanced, and can give rise to neoplastic transformations.

## 6. Conclusions

Induction of ROS by Cd at cellular level has been shown repeatedly [15] and the organ/cell-specific effects of ROS induced by Cd at different experimental conditions are reviewed here. The different experimental set-ups include differentiated cells at the whole animal level (including humans), tissue level, primary cell cultures and/or cell lines as well as non-differentiated cells. The appearance of ROS, depletion of scavengers, interference with antioxidant enzymes and/or damage to mitochondria results in loss of function or cell death in multiple organs. While Cd-induced ROS result in the degradation of Na/K pump function in kidney leading to dysfunctional transport, lipid peroxidation induced by Cd can be the detrimental cause of damage in bones. In general, it might be of interest to compare the Cd exposure levels *in vivo* to those *in vitro*, though comparisons between experiments should be done with caution. This is true in case of cell lines as they are generally more resistant to stress. Many studies use high concentrations of Cd or other test substances that might cause effects in cell lines, which could be irrelevant in terms of environmentally realistic exposures. Cadmium, in its carcinogenic role, activates oncogenes, inhibits tumour suppressor genes as well as affects signalling cascades. While Cd can interfere with DNA repair directly by replacing Zn in proteins involved in the repair, the appearance of 8-OHdG shows involvement of ROS in Cd-tumorogenesis. Epigenetic regulation induced by ROS has been demonstrated in studies and further investigations are needed to unravel the role of Cd and its interference with the epigenome. The last section of this review discusses stem cells that are highly resistant to multiple stressors. Stem cells defend themselves against Cd by being quiescent (keeping ROS levels low), thereby activating damage control systems (increasing the levels of MT and GSH) or by triggering apoptosis. Alternatively Cd-induced genomic instability may lead to neoplastic transformation and cancer. Taken together, a definite and important role for oxidative stress is evident in Cd-induced toxicity and pathogenesis, and the answer to the question, “where is the oxidative balance lost?” depends on a multitude of experimental and possible environmental conditions available for the cell and thereby organs.
